# B-cell immune dysregulation with low soluble CD22 levels in refractory seronegative myasthenia gravis

**DOI:** 10.3389/fimmu.2024.1382320

**Published:** 2024-04-22

**Authors:** Yuumi Okuzono, Shuuichi Miyakawa, Tatsuo Itou, Masaki Sagara, Masashi Iwata, Kei Ishizuchi, Koji Sekiguchi, Haruhiko Motegi, Munenori Oyama, Dnyaneshwar Warude, Yusuke Kikukawa, Shigeaki Suzuki

**Affiliations:** ^1^Oncology Drug Discovery Unit Japan, Research, Takeda Pharmaceutical Company Limited, Kanagawa, Japan; ^2^Department of Neurology, Keio University School of Medicine, Tokyo, Japan; ^3^Department of Neurology, The Jikei University School of Medicine, Tokyo, Japan

**Keywords:** myasthenia gravis, single-cell RNA-sequencing, immune dysregulation, B cell, soluble CD22

## Abstract

Myasthenia gravis (MG), primarily caused by acetylcholine receptor (AChR) autoantibodies, is a chronic autoimmune disorder causing severe muscle weakness and fatigability. In particular, seronegative MG constitutes 10%–15% of MG cases and presents diagnostic challenges especially in early-onset female patients who often show severe disease and resistance to immunosuppressive therapy. Furthermore, the immunopathology of seronegative MG remains unclear. Thus, in this study, we aimed to elucidate the pathogenic mechanism of seronegative MG using scRNA-seq analysis and plasma proteome analysis; in particular, we investigated the relationship between immune dysregulation status and disease severity in refractory seronegative MG. Employing single-cell RNA-sequencing and plasma proteome analyses, we analyzed peripheral blood samples from 30 women divided into three groups: 10 healthy controls, 10 early-onset AChR-positive MG, and 10 refractory early-onset seronegative MG patients, both before and after intravenous immunoglobulin treatment. The disease severity was evaluated using the MG-Activities of Daily Living (ADL), MG composite (MGC), and revised 15-item MG-Quality of Life (QOL) scales. We observed numerical abnormalities in multiple immune cells, particularly B cells, in patients with refractory seronegative MG, correlating with disease activity. Notably, severe MG cases had fewer regulatory T cells without functional abnormalities. Memory B cells were found to be enriched in peripheral blood cells compared with naïve B cells. Moreover, plasma proteome analysis indicated significantly lower plasma protein levels of soluble CD22, expressed in the lineage of B-cell maturation (including mature B cells and memory B cells), in refractory seronegative MG patients than in healthy donors or patients with AChR-positive MG. Soluble CD22 levels were correlated with disease severity, B-cell frequency, and RNA expression levels of CD22. In summary, this study elucidates the immunopathology of refractory seronegative MG, highlighting immune disorders centered on B cells and diminished soluble CD22 levels. These insights pave the way for novel MG treatment strategies focused on B-cell biology.

## 1 Introduction

Myasthenia gravis (MG), a chronic autoimmune disorder characterized by severe muscle weakness, is caused by autoantibodies that target neuromuscular junction proteins, damaging the postsynaptic muscle membrane and impairing signal transmission from motor neurons to the muscle (1). Patients with MG suffer from easy fatigability, which impairs their quality of life (QOL) (1). Despite major advancements in MG management, there remains a significant proportion of patients (approximately 20%) who are refractory to treatment (2, 3).

MG diagnosis is based on a detailed patient history, muscle testing for fatigability, electrodiagnostic testing for physiological decrement through repetitive nerve stimulation, and single-fiber electromyography (EMG) detection of neuromuscular junction inefficiency. The detection of autoantibodies against neuromuscular junction proteins in the serum, most commonly acetylcholine receptor (AChR) or muscle-specific tyrosine kinase (MuSK), and rarely low-density lipoprotein receptor-related protein 4 (LRP4), is crucial for diagnosis (1). Initially, seronegative MG was defined as the absence of anti-AChR antibodies in the patient’s serum (4). Seronegative MG is characterized by the absence of antibodies against AChR, MuSK, and LRP4 antigens in commercially available serological tests (1). Ten to fifteen percent of patients with MG are seronegative (1). The diagnosis of seronegative MG is sometimes challenging owing to the absence of detectable autoantibodies, and its immunopathogenesis is less studied compared with that of AChR- and MuSK-positive MG. In addition, seronegative MG comprises various clinical phenotypes, including ocular and generalized, or early and late onset (cutoff age of 50 years). Refractory MG is defined as MG in which the symptoms cannot be well controlled, or therapy for adequate control cannot be tolerated due to side effects and/or burdens, even when therapy with two or more immunosuppressive oral agents or with rescue therapy is provided (2). Refractory early-onset seronegative MG, especially in early-onset female patients, is characterized by severe disease and resistance to immunosuppressive therapy (3, 5, 6).

Regulatory T cells (Tregs) are involved in AChR-positive MG (7–9) and B-cell-related rituximab sensitivity (10–12), from the activation of the immune response by Treg dysfunction and Treg reduction for the promotion of autoantibody production which contributes to the exacerbation of the MG pathogenesis. Several associations between B cells and MG, which has detectable antibodies, have also been reported (13–15). However, the overall mechanism of seronegative MG pathogenesis, mediated by immune cells, remains unclear. The combination of single-cell RNA-sequencing (scRNA-seq) and computational analysis is a revolutionary technology that enables simultaneous immune profiling of each cell by applying comprehensive gene expression information to individual immune cells, and it elucidates novel immune pathologies of autoimmune diseases (16–18). The use of scRNA-seq is expected to support our understanding of pathologies to the disease where the mechanism has been unclear, such as seronegative MG. However, to the best of our knowledge, only a handful of scRNA-seq studies have been conducted in MG to date (10, 19), and previous studies were relatively small-scale analyses of the population.

Since no studies have been conducted in patients with refractory seronegative MG, the objective was to elucidate the pathogenic mechanism of seronegative MG using scRNA-seq analysis and plasma proteome analysis. In particular, we aimed to investigate the immune dysregulation in refractory seronegative MG to improve the QOL for refractory patients. The results will help better understand the development of MG and provide a strategy for the development of therapeutics based on B-cell biology.

## 2 Materials and methods

### 2.1 Patients and healthy controls

We enrolled early-onset (disease onset, <50 years) and generalized female patients with AChR-positive and seronegative MG who were examined at the Keio University Hospital (Tokyo, Japan). Patients were diagnosed with seronegative MG if they had the following attributes: (i) a clinical history of fatigable weakness; (ii) objective fatigable weakness on physical examination; (iii) undetectable anti-AchR, anti-MuSK, and anti-LRP4 antibodies using radioimmunoassays and luciferase immunoprecipitation; and (iv) electrophysiological abnormalities, either ≥10% decrease in the compound muscle action potential amplitudes at 3-Hz repetitive stimulation or motor unit potential variation or abnormal single-fiber EMG in at least one cranial or limb muscle. Single-fiber EMG was required to be abnormal in those without an identified decrement when evaluating the orbicularis oris, frontalis, or extensor digitorum communis muscles, where defined abnormalities have been established with normal reference values. Clinical status and severity were determined according to the recommendations of the Myasthenia Gravis Foundation of America (MGFA) (20). The current MG status was evaluated using three clinical scales: the MG- Activities of Daily Living (ADL), the MG composite (MGC), and the revised 15-item MG-QOL scales (21–23). Blood samples were obtained from seronegative MG patients under the immunotherapy, whereas they were obtained from AChR-positive without the immunotherapy.

Furthermore, we enrolled 10 healthy female controls. Individuals with ICD-10 diagnostic codes for autoimmune diseases, endocrine disorders including diabetes and thyroid disease, malignant and non-malignant neoplasms, any musculoskeletal disorder, and all nervous system disorders were excluded. For immunosuppressive treatment, we selected AChR-positive patients who had never received steroids, other immunosuppressive agents, intravenous immunoglobulin (IVIg), or plasmapheresis during their entire clinical course. In contrast, we included patients with refractory seronegative MG who suffered from severe diseases and received a combination of various immunosuppressive regimens.

All clinical information was collected after the patients provided written informed consent. All study protocols were approved by the Institutional Review Board of Keio University (#2020-0074). These clinical investigations were conducted in accordance with the principles of the Declaration of Helsinki.

### 2.2 Peripheral blood mononuclear cell and plasma preparation

**Figure 1:**
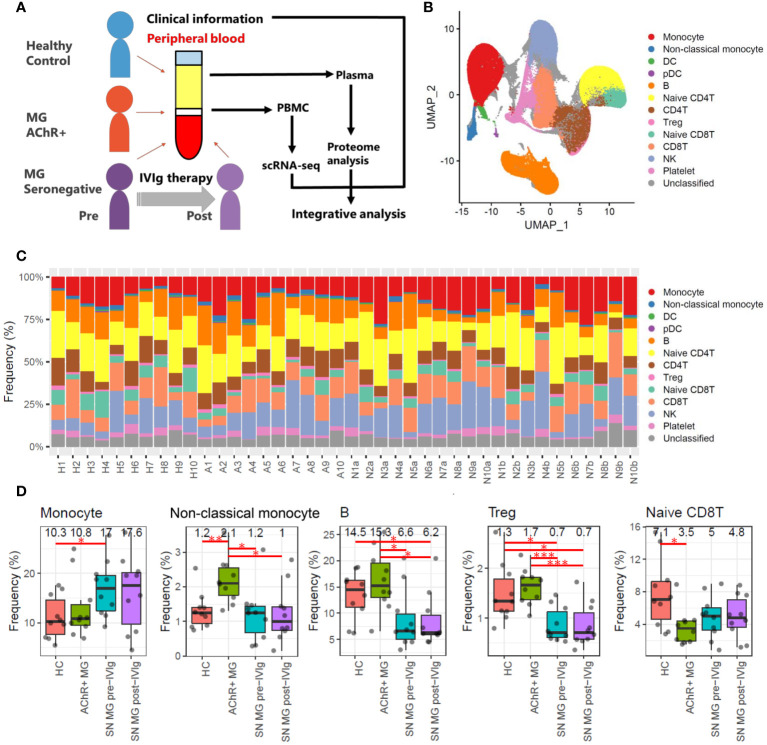
Overall study design and frequency profiling for each cell type in scRNA-seq data. **(A)** Overview of this study integrating scRNA-seq of PBMCs, plasma proteome, and clinical information for healthy controls (HCs), patients with acetylcholine receptor-positive (AChR+) myasthenia gravis (MG), and seronegative (SN) MG pre-/post-intravenous immunoglobulin (IVIg)-treated patients. **(B)** Uniform Manifold Approximation and Projection (UMAP) showing 13 identified cell types: monocytes (CD14 positive monocytes), non-classical monocytes (CD16 positive monocytes), dendritic cells (DCs), plasmacytoid dendritic cells (pDCs), B cells, naïve CD4 T cells, CD4 T cells, regulatory T cells (Tregs), naïve CD8 T cells, CD8 T cells, natural killer (NK) cells, platelets, and unclassified cells in all samples (HCs, AChR+ MG, and SN MG pre-/post-IVIg treated patients). **(C)** Frequencies of each cell type for each sample. The frequency is presented as the percentage of each cell type when the total number of cells in each sample is 100%. Numbers beginning with H refer to healthy control, numbers beginning with A refer to patients with AChR+ myasthenia gravis, numbers beginning with N and ending with a refer to SN MG pre-intravenous immunoglobulin (IVIg)-treated patients, and numbers beginning with N and ending with b refer to SN MG post-intravenous immunoglobulin (IVIg)-treated patients. This number corresponds to the patient ID in **Supplementary Table 3**. **(D)** Boxplots of cell frequencies of cell types that showed significant differences in the Tukey’s HSD test (P < 0.05). Numerical values show the median for each group. Red lines and asterisks show significant combinations in the Tukey’s HSD test (*P < 0.05, **P < 0.005, and ***P < 0.0005).

Human peripheral blood samples were collected from anonymous healthy donors and patients with MG at Keio University Hospital (**Figure 1A**). PBMCs were separated using a LeucoSep tube filled with Ficoll-Paque-plus (Greiner Bio-One, Kremsmünster, Austria), according to the manufacturer’s instructions. Briefly, blood samples were transferred into tubes and centrifuged at 1000 ×*g* for 10 min at room temperature. PBMCs from the interfacial layer were collected, washed with 1% bovine serum albumin (BSA)/phosphate-buffered saline (PBS), resuspended in 1% BSA/PBS, and filtered through 40-μm FLOWMI Cell Strainers (Bel-Art Products, South Wayne, NJ, USA). scRNA-seq analysis was performed on cells resuspended in 1% BSA/PBS at a concentration of 1 × 10^6^ cells/ml. PBMCs contaminated with red blood cells (RBCs) were subjected to RBC lysis using RBC lysis solution (Miltenyi Biotec, Bergisch Gladbacblooh, Germany). RBCs were removed by incubating PBMCs in 10 volumes of 10X RBC lysis solution diluted with double-distilled H_2_O for 10 min at room temperature. To remove the lysis buffer, PBMCs were washed with 1% BSA/PBS. Heparin plasma was isolated from the blood of the same patient in a different tube than that used for PBMC collection through centrifugation at 3000 rpm for 10 min at 4°C.

### 2.3 scRNA-seq library preparation and sequencing

scRNA-seq was performed using the 10x Genomics platform. Single-cell suspensions (10,000 cells) were loaded onto a chip for GEM (Gel Beads-in-emulsion) generation using the 10x Genomics Chromium controller, and DNA libraries were prepared using Chromium Next GEM Single Cell 3’ Reagent Kits v3.1 (10x Genomics, Pleasanton, CA, USA), according to the manufacturer’s instructions. Quality control of the prepared libraries was performed using an Agilent 2100 Bioanalyzer before sequencing (Santa Clara, CA, USA). Gene expression libraries were sequenced on the DNBSEQ platform at a depth of 50,000 reads per cell to generate binary-base call (bcl) files.

### 2.4 scRNA-seq data normalization

Primary analysis was conducted using the 10x Genomics Cell Ranger version 3 pipeline. The bcl files were converted to FASTQ format using Cell Ranger “counts” software. FASTQ data were filtered and mapped to the GRCh38 reference genome. Secondary analysis was conducted using R version 4.0.2 with the Seurat version 3 package (24, 25). Data on low-quality cells and doublet cells were excluded using the Seurat and DoubletFinder packages (26). These counts were normalized using a global-scaling normalization method with the Seurat function “LogNormalize” and log-transformed using the Seurat function “NormalizeData.” The variable features were selected by direct modeling of the mean-variance relationship inherent using the Seurat function “FindVariableFeatures,” and these features were used to choose integration features using the Seurat function “SelectIntegrationFeatures.” The integration features were applied to the linear transformation and used for principal component analysis (PCA) using the Seurat functions “ScaleData” and “RunPCA,” respectively. Using reciprocal PCA, the top 50 principal components were used to identify anchors. Anchors were used to integrate datasets using the Seurat function “IntegrateData” (25). All single-cell RNA-seq data were deposited in the Gene Expression Omnibus (GEO) repository at http://www.ncbi.nlm.nih.gov/geo (accession number: GSE227835).

### 2.5 scRNA-seq analysis

Integrated normalized data were subjected to linear transformation and PCA using the Seurat functions “ScaleData” and “RunPCA.” The important principal components were determined using the elbow plot, which plotted the standard deviations of the principal components and was adopted to visualize the Uniform Manifold Approximation and Projection (UMAP) using the Seurat functions “ElbowPlot” and “RunUMAP.” Important principal components were also used to identify cell types. First, the similarities between each cell were calculated by a KNN graph based on the Euclidean distance in PCA space, and the edge weights between any two cells were based on the shared overlap in their local neighborhoods by Jaccard’s similarity using the Seurat function “FindNeighbors.” Next, the cells were divided into clusters by clustering the similarity between the cells using the Louvain algorithm and the Seurat function “FindClusters.” Finally, the cell type in each cluster was identified by examining the cell type-specific marker expression profiles in each cluster. Differentially expressed genes (DEGs) between the groups in each cell type were identified with the non-parametric Wilcoxon rank-sum test using the Seurat function “FindMarkers.” Gene expression levels in each cluster were visualized in violin plots using the Seurat function “VlnPlot.” The differences in frequencies of each cell type between the groups were identified with Tukey’s honestly significant difference (HSD) using the “TukeyHSD” function in the stats (R default) package. Correlation tests were conducted using the “cor.test” function in the stats (R default) package. The visualization of scatter plots, boxplots, and bar plots was performed in R using the ggplot2 package (27).

### 2.6 Plasma protein measurements

In total, 402 human proteins related to inflammation in plasma were measured simultaneously using the Olink Target 96 Inflammation and Olink Explore 384 Inflammation panels (Olink Proteomics, Uppsala, Sweden). The measured proteins are listed in **Supplementary Table 1**. The measurement was based on proximity extension assay (PEA) technology (https://www.olink.com). In brief, a matched pair of antibodies linked to distinct oligonucleotides (proximity probes) bind to their respective protein targets. Only when the two probes are in close proximity to one another do they hybridize and form double-stranded DNA. Finally, the complex was detected and quantified by quantitative real-time PCR for the Olink Target 96 Inflammation panel or next-generation sequencing (NGS) for the Olink Explore 384 Inflammation panel. Data are presented as normalized protein expression (NPX) values and Olink Proteomics arbitrary units on a log_2_ scale. NPX values were calculated using CT values (quantitative real-time PCR) and the number of matched counts. Therefore, an NPX value represents relative expression rather than an absolute protein level. All plasma proteomic data are presented in **Supplementary Table 2**.

### 2.7 Plasma proteome analysis

For the proteins analyzed, all items measured by NGS were selected, whereas for items measured only by real-time PCR, only samples with more than 50% of the samples above the detection limit were selected. Differentially expressed proteins (DEPs) between the groups were identified using Tukey’s HSD test with the “TukeyHSD” function in the stats (R default) package. Volcano plots were generated in R using the ggplot2 and ggrepel packages (27, 28). The visualization of the scatter plot and boxplot was performed in R using the ggplot2 package (27). Correlation tests were conducted using the “cor.test” function in the stats (R default) package.

### 2.8 Suppression assay of Tregs

The suppressor activity of expanded Tregs was analyzed using co-culture assays. Human PBMCs (Lonza) were labeled with Cell Trace Violet (Thermo Fisher Scientific, Waltham, MA, USA), according to the manufacturer’s protocol. Expanded Tregs were cultured with cell trace violet-labeled PBMCs at different ratios together with Dynabeads human T-activator CD3/CD28 (Thermo Fisher) for 3 days. Proliferated PBMCs were stained with anti-CD4-PEcy7 (BioLegend, San Diego, CA, USA). The proliferation of CD4^+^ T cells was analyzed using a BD FACSAria (BD Biosciences, San Jose, CA, USA). The sigmoid curves of Treg suppression functions were visualized in R using the ggplot2 package (27) and the nls2 package (29).

### 2.9 Isolation and expansion of Tregs

CD4^+^ T cells were isolated from human PBMCs using CD4 microbeads (Miltenyi Biotec), according to the manufacturer’s protocol. Purified CD4T cells were cultured overnight in X-VIVO 15 serum-free hematopoietic cell media (Lonza, Basel, Switzerland) supplemented with 5% fetal bovine serum (Thermo Fisher Scientific). CD4 T cells were labeled with CD4-PEcy7 (BioLegend), CD127-BV510 (BioLegend), CD45RA-FITC (BioLegend), CXCR5-AF647 (BioLegend), and CD25-BV421 (BioLegend). Fluorescent-activated cell sorting (FACS) of naïve Tregs (CD4^+^, CD25 high, CD127 low, and CD45RA^+^) was performed using BD FACSAria Fusion (BD Biosciences). Purified Tregs were stimulated using the Dynabeads human Treg expander (Thermo Fisher) at a 4:1 bead-to-cell ratio. The TNFR2-agonist (Hycult Biotech, Uden, Netherlands) and rapamycin (LKT Laboratories, Inc., St. Paul, MN, USA) were added at the start of the culture. On day 2, human IL-2 (Miltenyi Biotec) was added. The culture medium was replenished every 2 or 3 days. On day 9, the cells were restimulated with the Dynabeads human Treg expander in a 1:1 bead-to-cell ratio. On day 13, the cells were harvested, counted, and analyzed. The proliferation rate was calculated as the number of Tregs on day 13 divided by the number of isolated Tregs from PBMCs. The differences in proliferation rate between the groups were identified with Tukey’s HSD test using the “TukeyHSD” function in the stats (R default) package and visualized in R as the boxplot using the ggplot2 package (27).

### 2.10 Statistics and reproducibility

Statistical analyses were performed with Microsoft R open software (version 4.0.2). DEGs in scRNA-seq were identified using the non-parametric Wilcoxon rank-sum test and corrected for multiple testing using Bonferroni’s test. DEPs in the plasma proteome and the differences in the frequencies of each cell type between the groups were identified using Tukey’s HSD for multiple testing. Correlation tests were conducted using Pearson’s correlation coefficient.

## 3 Results

### 3.1 Clinical features

Peripheral blood samples were obtained from 30 individuals in three groups: 10 healthy controls (HCs), 10 AChR-positive patients with MG, and 10 seronegative patients with MG (**Table 1**; **Supplementary Table 3**). The participants were all women, aged 26–60 years. The onset age of MG was similar in the AChR-positive and seronegative MG groups, indicating early-onset MG. A total of 20 patients had a generalized form of MG. To exclude the effects of immunotherapy on profiling immune changes, we selected 10 AChR-positive patients with MG who had never received immunotherapy during the entire course, despite the fact that four patients underwent thymectomy. Thymectomy was performed soon after the diagnosis of MG, on average of 12 years before the sample collection. Ten AChR-positive patients with MG had mild involvement according to the clinical classification and favorable disease control by postintervention status based on the recommendations of the MGFA.

**Table 1:** Clinical features of 30 participants.

	Healthy control (n=10)	AChR-positive MG (n=10)	Seronegative MG (n=10)
Age, years	41.3 ± 10.7	45.2 ± 11.3	43.1 ± 9.8
Female sex	10 (100%)	10 (100%)	10 (100%)
Onset age, years		33.9	31.8
Disease duration, years		11.3	11.2
MGFA classification			
Mild generalized		8 (80%)	0 (0%)
Moderate generalized		2 (20%)	8 (80%)
Severe generalized		0	2 (20%)
Bulbar symptoms		4 (40%)	10 (100%)
Myasthenic crisis		0 (0%)	0 (0%)
AChR Ab positive		10 (100%)	0 (0%)
Treatment			
Thymectomy		4 (40%)	1 (10%)
Prednisolone		0 (0%)	10 (100%)
Calcineurin inhibitors		0 (0%)	10 (100%)
IVIg		0 (0%)	10 (100%)
Plasmapheresis		0 (0%)	4 (40%)
Postinterventional status			
Minimal manifestations		5 (50%)	0 (0%)
Improved		4 (40%)	3 (30%)
Unchanged		1 (10%)	7 (70%)
Clinical scales*			
MGC		4.2 ± 3.1	21.1 ± 3.8/16.8 ± 2.9
MG-ADL		3.2 ± 2.0	14.0 ± 1.3/10.7 ± 1.3
15-item MG-QOL		5.8 ± 4.6	20.1 ± 5.0/15.4 ± 3.8

*Clinical scales were evaluated pre- and post-intravenous immunoglobulin (IVIg) treatment in seronegative myasthenia gravis (MG) patients (indicated as pre- and post- IVIg).

AChR, acetylcholine receptor; MGC, MG composite scale; MG-ADL, MG-Activities of Daily Living.

In contrast, 10 patients with seronegative MG had moderate-to-severe generalized symptoms, including difficulties in speaking and swallowing. All patients received a combination of prednisolone (a daily dose of 8.1 mg/day) and calcineurin inhibitors, including tacrolimus (n = 8) and cyclosporine (n = 2). Only one patient with seronegative MG underwent thymectomy, although the general consensus showed thymectomy was not effective for seronegative MG (30). In addition, they required rescue treatment, such as IVIg and plasmapheresis, at least twice every year. Regarding plasmapheresis, plasma exchange was performed on four seronegative patients with MG. In these cases, the interval between the final plasma exchange and sample collection was over 2 years. Based on the disease severity and treatment profiles, patients with seronegative MG were regarded as having “refractory” MG. To further evaluate the association between immune changes and disease severity, we collected chronological samples from 10 patients with refractory seronegative MG pre- and post-IVIg treatment. The effects of IVIg usually appeared 2−3 weeks after treatment and lasted until 2−3 months after treatment in patients with seronegative MG (31). The clinical improvement with immunotherapy suggested that patients with seronegative MG did not have congenital MG. We collected additional blood samples 3 months after IVIg treatment, when clinical improvement was confirmed by proper severity measurement using the MGC scale. Additionally, we used patient-oriented evaluation methods, including the MG-ADL scale and a revised 15-item MG-QOL scale.

### 3.2 scRNA-seq revealed numerical changes in immune cells in patients with MG

We used the principal components of the integrated data to generate a unified UMAP embedding space. Graph-based clustering was performed using the Louvain algorithm, and each cluster was annotated with cell type markers (**Supplementary Figures 1**, **2**). The integrated scRNA-seq data were classified into 13 clusters, and 11 cell types (monocytes, non-classical monocytes, dendritic cells (DCs), plasmacytoid dendritic cells (pDCs), B cells, naïve CD4 T cells, CD4 T cells, regulatory T cells (Tregs), naïve CD8 T cells, CD8 T cells, and natural killer (NK) cells) were selected for further analysis (**Figure 1B**).

To investigate the immune profiles of each group, we first calculated the immune cell type frequencies of HC, AChR-positive MG, and seronegative MG in pre- or post-IVIg treatment (**Figure 1C**). This analysis indicated that five (monocytes, non-classical monocytes, B cells, Tregs, and CD8Ts) out of the 11 cell types showed significant differences. The number of monocytes was higher in the seronegative MG group than in the HC group, and the number of non-classical monocytes was higher in the AChR-positive MG group than in the HC or seronegative MG groups (**Figure 1D**). Notably, the seronegative MG group had fewer B cells and Tregs than the HC or AChR-positive MG groups, and the AChR-positive MG group had fewer naïve CD8Ts than that in the HC group (**Figure 1D**). However, no significant differences between pre- and post-treatment with IVIg therapy were observed in the cell type frequencies of all cell types in patients with seronegative MG. Likewise, no significant differences were observed in cell type frequencies in any of the evaluated cell types between patients with seronegative MG with and without plasmapheresis. In addition, no significant differences were observed in cell type frequencies in any of the evaluated cell types between patients with AChR-positive MG with and without thymectomy (**Supplementary Table 4**). Therefore, these results revealed that the immunological profiles of AChR-positive MG and seronegative MG were clearly different, and IVIg treatment did not result in a statistically significant change in the frequency of immune cell types in patients with seronegative MG.

### 3.3 Frequencies of several immune cells were correlated with disease severity

**Figure 2:**
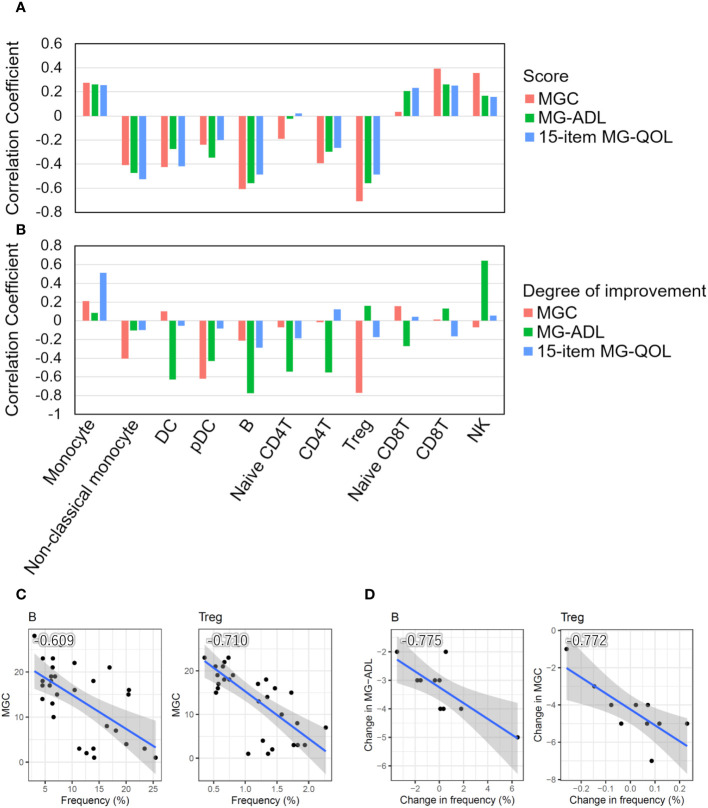
Correlation between cell type frequencies and clinical information. **(A)** Bar plots showing Pearson’s correlation coefficients between the frequencies of 11 cell types and disease severity scores (MGC and MG-ADL) and the 15-item MG-QOL. **(B)** Bar plots showing Pearson’s correlation coefficients between the degrees of disease alleviation through intravenous immunoglobulin (IVIg) administration in patients with seronegative (SN) myasthenia gravis (MG) and the frequency of changes in each cell type. **(C)** Scatter plots with a particularly significant correlation between the frequency of cell type and disease severity score in MG patients. **(D)** Scatter plots with a particularly significant correlation between the degrees of disease alleviation by IVIg treatment in patients with SN MG and the frequency changes. The blue line indicates the regression line, and the gray zone indicates the 95% confidence interval. DC, dendritic cell; pDC, plasmacytoid DC; Treg, regulatory T cell; NK, natural killer cell; MGC, MG composite scale; MG-ADL, MG-Activities of Daily Living.

We investigated the relationship between the frequencies of each cell type and disease severity. The frequencies of B cells and Tregs were negatively correlated with MGC, MG-ADL, and the 15-item MG-QOL. The frequency of non-classical monocytes was negatively correlated with the 15-item MG-QOL (P < 0.0001) (**Figure 2A**).

In addition, we investigated the relationship between the degree of disease alleviation by IVIg treatment in patients with seronegative MG and the frequency of changes in each cell type. The differences in disease severity scores between pre- and post-IVIg treatment in patients with seronegative MG were used to calculate the degree of disease improvement, while the difference in frequencies for each cell type was used to calculate the frequency changes in each cell type. The frequencies of DCs, pDCs, B cells, naïve CD4T cells, CD4T cells, and Tregs increased as the disease was alleviated, whereas the frequencies of monocytes and NK cells decreased as the disease progressed (**Figure 2B**). Therefore, the change in frequency of B cells and Tregs was demonstrated to be associated with both disease severity and the degree of disease alleviation by IVIg treatment in patients with seronegative MG (**Figures 2C, D**).

Taken together, these results suggest that disease severity increased as the number of Tregs and B cells decreased, and symptoms were alleviated as the number of Tregs and B cells increased after IVIg treatment, indicating that Tregs and B cells are closely related to clinical scores.

### 3.4 Gene expression differences were not observed in immune cells of patients with MG

We observed numerical changes in immune cell types in patients with MG and a correlation between the difference and disease severity; therefore, we investigated the relationship between MG and qualitative changes in immune cells. To understand the overall qualitative changes in each group, DEG analysis was performed using scRNA-seq data. We performed differential expression analysis of scRNA-seq data between AChR-positive MG vs. HC, seronegative MG vs. HC, and AChR-positive MG vs. seronegative MG, and then isolated the DEGs in 7 cell types (**Supplementary Table 5**). However, very few isolated DEGs were identified in each comparison (**Table 2**). In addition, we compared pre- and post-IVIg treatment in patients with seronegative MG and isolated DEGs; however, there were no differences (**Table 2**). Surprisingly, these results showed no discernible differences in gene expression in MG immune cells.

**Table 2:** List of the number of differentially expressed genes (DEGs).

Cell type	AChR-positive MG vs. HC	Seronegative MG vs. HC	Seronegative MG vs. AChR-positive MG	Seronegative MG pre-IVIg vs. post-IVIg
**Monocyte**	0	1	1	0
**Non-classical monocyte**	0	2	1	0
**DC**	0	0	0	0
**pDC**	0	1	1	0
**B**	2	16	6	0
**Naïve CD4T**	0	0	0	0
**CD4T**	0	0	0	0
**Treg**	1	0	0	0
**CD8T**	0	0	2	0
**Naïve CD8T**	0	0	0	0
**NK**	0	1	0	0

MG, myasthenia gravis; HC, healthy control; IVIg, intravenous immunoglobulin; AChR, acetylcholine receptor; DCs, dendritic cells; pDCs, plasmacytoid DCs; Tregs, regulatory T cells; NK, natural killer cells.

### 3.5 Tregs in patients with MG showed no functional abnormalities

**Figure 3:**
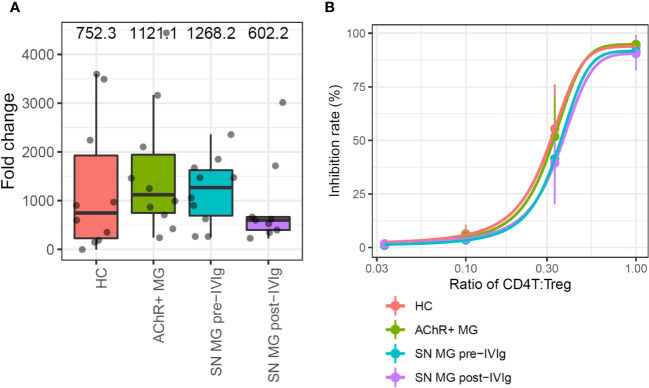
*In vitro* assays for the comparison of regulatory T cell (Treg) functions in each group. **(A)** Box plots showing the proliferation rate as fold changes of cell counts between pre- and post-expansion for each group. Numerical values show the median for each group (P value is not stated because there were no statistically significant differences). **(B)** Sigmoid curve plots showing Treg-suppressive functions measured based on the inhibition rate of cell division of CD4T cells co-cultured with Tregs. Error bars indicate the standard deviation (P value is not stated because there were no statistically significant differences). MG, myasthenia gravis; HC, healthy control; AChR+, acetylcholine receptor-positive; SN, seronegative.

To investigate the function of Tregs and B cells, which were significantly highlighted in immune cell type frequency analysis and disease severity analysis, we first examined the proliferation ability and suppressive function of Tregs isolated from the PBMCs of patients with MG. The proliferation ability of each group—HC, AChR-positive MG, and seronegative MG with pre-IVIg treatment and post-IVIg treatment—was measured by fold changes of cell counts between pre- and post-expansion, but no significant differences were observed between any of the groups (**Figure 3A**).

The suppressive function was measured from the inhibition rate of cell division of CD4T cells, which were co-cultured with Tregs; the functions were compared using Tukey’s HSD test. Since the number of Tregs which were isolated from PBMC were limited, 13-days *in vitro* Treg expansion was required to perform suppressive function study. In this assay, Tregs suppressed CD4T cell proliferation in a dose-dependent manner, but no difference was observed in the suppression abilities of Tregs among the groups (**Figure 3B**). In addition, we conducted a subpopulation analysis for Tregs to investigate whether there were qualitative abnormalities based on the scRNA-seq data and subpopulation-specific marker expression profiles; Tregs did not exhibit a distinct subpopulation (**Supplementary Figure 3**. These results suggested that patients with more severe MG had fewer Tregs, but the Tregs did not exhibit any functional abnormalities.

### 3.6 Memory B cells were the dominant subpopulation of B cells in patients with seronegative MG

**Figure 4:**
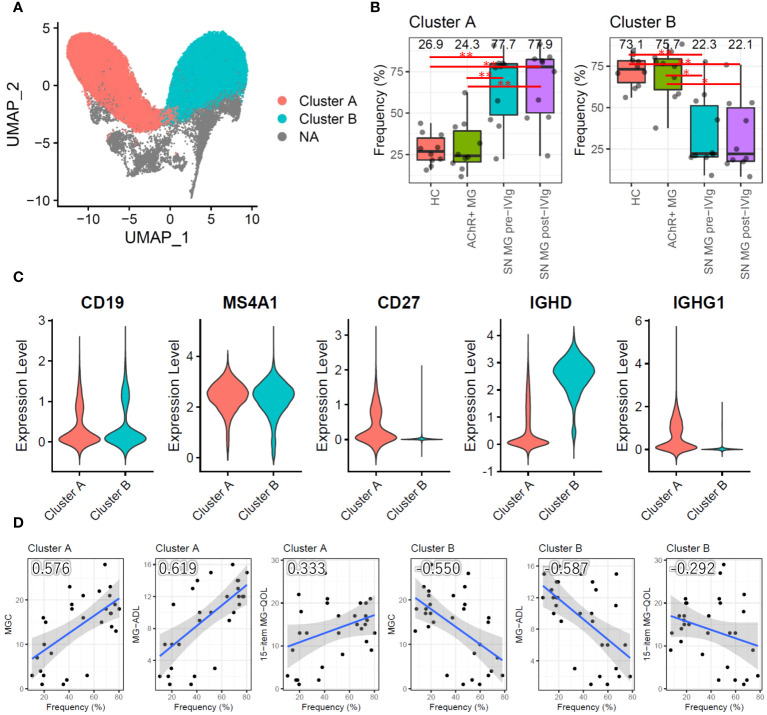
Subpopulation analysis of B cells. **(A)** Uniform Manifold Approximation and Projection (UMAP) showing two B-cell subpopulation clusters in all samples (HCs, AChR-positive MG, and seronegative MG pre-/post-IVIg treated patients). **(B)** Box plots showing the cell frequencies of each B-cell subpopulation cluster in each group. Numerical values show the median for each group. Red lines and asterisks show statistically significant combinations based on Tukey’s HSD test (*P < 0.05 and **P < 0.005). **(C)** Violin plots showing the gene expression levels of B-cell marker genes for each B-cell subpopulation cluster. **(D)** Scatter plots between the frequency of each B-cell subpopulation cluster and disease severity score with Pearson’s correlation coefficient. Numerical values show the correlation coefficient using 30 MG patient samples (AChR-positive MG and seronegative MG pre-/post-IVIg treated patients). The blue line indicates the regression line, and the gray zone indicates the 95% confidence interval. MG, myasthenia gravis; AChR+, acetylcholine receptor-positive; SN, seronegative; IVIg, intravenous immunoglobulin.

We conducted a qualitative analysis to better understand the role of B cells in MG. Instead of a functional assessment of B cells, we performed a subpopulation analysis to determine the differences among the groups. The primary UMAP clustering did not show a B-cell subpopulation (**Figure 1B**); however, B-cell-focused subpopulation analysis revealed two B-cell subpopulation clusters, named clusters A and B (**Figure 4A**). When compared with those in the HC and AChR-positive MG groups, cluster A had a significantly higher frequency, and cluster B had a significantly lower frequency in the seronegative MG group (P < 0.01) (**Figure 4B**). To identify the subtype of B cells in clusters A and B, we investigated the expression of the marker gene for B-cell subtyping. Cluster A highly expressed IgG genes (*IGHG1*, *IGHG2*, *IGHG3*, and *IGHG4*) as marker genes for memory B cells (CD19^+^CD20^+^CD27^+^IgG^+^), whereas cluster B showed high expression of the IgD gene (*IGHD*) as the marker gene for naïve B cells (CD19^+^CD20^+^CD27^-^IgD^+^) (**Figure 4C**). Memory B cells were the dominant B-cell subpopulation in seronegative MG patients, while naïve B cells were the dominant B-cell subpopulation in the HC and AChR-positive MG groups. We investigated the relationships between the frequencies of clusters A and B and disease severity using correlation analysis. Cluster A showed a positive correlation, and cluster B showed a negative correlation with disease severity (**Figure 4D**). Integrating previous results, we found that the cell frequency of pan-B cells was reduced in patients with MG, but the percentage of memory B cells was higher in seronegative MG than in AChR-positive MG.

### 3.7 Plasma soluble CD22 levels decreased in patients with seronegative MG

We analyzed plasma proteome data, which enabled the detection of 402 human proteins in the inflammation panels 92 and 384 (**Supplementary Table 1**).

**Figure 5:**
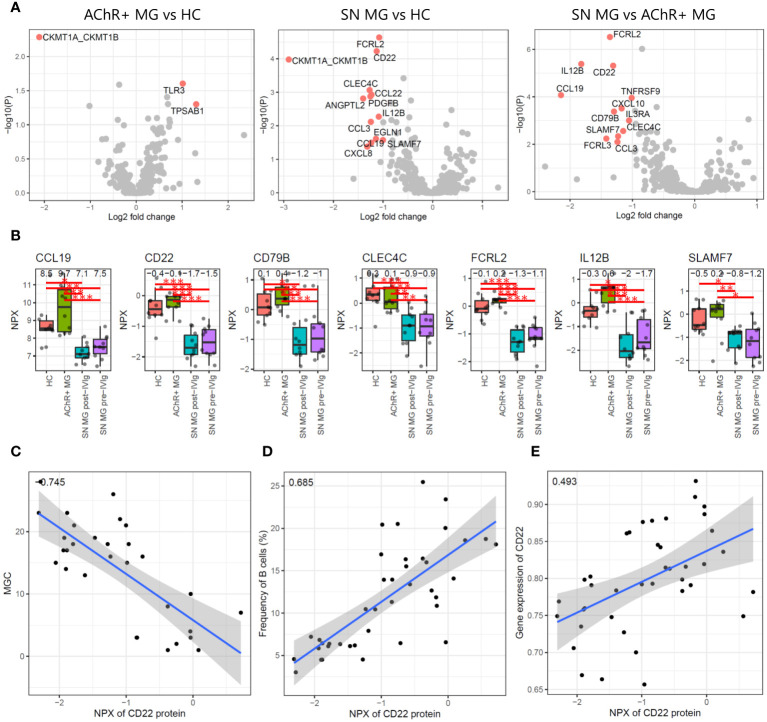
Plasma proteome analysis. **(A)** Volcano plots showing -log_10_ P-values and log_2_ fold changes from differential expression analysis between each combination. The red dots indicate the differentially expressed proteins (DEPs), and the protein names are shown. **(B)** Box plots showing expression levels of seven proteins that were isolated as DEPs with seronegative MGs compared to those in HC and AChR-positive MG. Numerical values show the median for each group. Red lines and asterisks show statistically significant combinations based on Tukey’s HSD test (*P < 0.05, **P < 0.005, and ***P < 0.0005). **(C)** Scatter plot between the expression levels of CD22 protein and the disease severity score by MGC with Pearson’s correlation coefficient. Numerical values show the correlation coefficient using 30 MG patient samples (AChR-positive MG and seronegative MG pre-/post-IVIg treated patients). **(D)** Scatter plot between the expression levels of CD22 protein and the frequency of B cells using Pearson’s correlation coefficient. Numerical values show the correlation coefficient using 30 MG patient samples. **(E)** Scatter plot between the expression levels of CD22 protein and the expression levels of CD22 genes based on the scRNA-seq data with Pearson’s correlation coefficient. Numerical values show the correlation coefficient using 30 MG patient samples. The blue line indicates the regression line, and the gray zone indicates the 95% confidence interval. MG, myasthenia gravis; HC, healthy control; IVIg, intravenous immunoglobulin; AChR, acetylcholine receptor.

We first conducted differential expression analysis between HC, AChR-positive MG, and seronegative MG and isolated DEPs (**Figure 5A**). Comparing AChR-positive MG and HC, TLR3 and TPSAB1 were upregulated and CKMT1A/CKMT1B were downregulated in AChR-positive MG (**Figure 5A**). Comparing seronegative MG and HC or AChR-positive MG, CCL19, CD22, CD79B, CLEC4C, FCRL2, IL12B, and SLAMF7 were downregulated in seronegative MG (**Figure 5A**). We also compared pre- and post-treatment with IVIg therapy in seronegative MG and found no DEPs (**Supplementary Figure 4**). The number of identified DEPs was higher in the comparison between seronegative MG and HC or seronegative MG and AChR-positive MG than that in the comparison between AChR-positive MG and HC.

Seven of 402 proteins were isolated as DEPs with seronegative MGs compared with both HC and AChR-positive MG (**Figure 5B**). To evaluate whether these seven proteins showed a correlation with disease severity, we performed a correlation analysis of seven DEPs with disease severity, immune cell frequency, and mRNA expression levels using Pearson’s correlation coefficient and the correlation test, respectively. Soluble CD22 protein was negatively correlated with disease severity and positively correlated with B-cell frequency and CD22 mRNA expression levels, all of which were statistically significant (P < 0.001) (**Figures 5C–E**). None of the other six DEPs showed significant correlations with any of the combinations.

Decreased B-cell numbers and low plasma levels of soluble CD22 were observed in patients with refractory seronegative MG. Notably, CD22 was expressed in the B-cell maturation lineage, including mature B cells and memory B cells. Therefore, B-cell-related events occurred in patients with refractory seronegative MG, resulting in decreased soluble CD22 levels and B-cell numbers.

## 4 Discussion

MG, including seronegative MG, is heterogeneous in terms of symptom presentation and pathophysiology, given that different proteins of the neuromuscular junction can be targeted. Seronegative MG is more common in ocular MG than in generalized MG; however, generalized seronegative MG may require special attention for refractory courses, especially in early-onset and female patients (3, 5, 6). The patients with refractory seronegative MG in this study were all women, with early-onset moderate-to-severe generalized forms. Their muscle weakness of the limbs, neck muscles, and bulbar symptoms was significantly impaired using appropriate clinical scores including MG-ADL, MG composite, and revised 15-item MG-QOL. All patients with refractory seronegative MG were treated with a combination of prednisolone and immunosuppressive agents and rescue therapy consisting of IVIg and/or intravenous steroid pulse therapy. All patients with refractory seronegative MG responded positively to rescue therapy, but the effects lasted several months. Therefore, they needed a course of IVIG therapy at least twice a year. These clinical features suggest a homogeneous subset of refractory seronegative MG cases. The Japan MG registry survey in 2021 revealed that 11% of the 1710 patients with MG had generalized MG and seronegative MG and were predominantly women, who mostly presented with severe refractory symptoms compared with patients with AChR-positive MG (3). Since number of samples was limited in the scRNA-seq study, we included the female MG patients alone to minimize a gender bias. Tomschik et al. reported that patients with seronegative MG were resistant to immunosuppressive therapy and had poor outcomes (5). A German multicenter study showed that myasthenic crises in seronegative MG affected younger patients after a longer period of disease (6).

Tregs directly or indirectly suppress effector T cells that mediate autoimmune responses by producing suppressive cytokines such as TGF-β and IL-10 or by suppressing antigen-presenting cells via CTLA4 (32). Owing to their strong suppressive ability, the adoptive transfer of autologous Tregs has been reported to suppress experimental autoimmune MG in rats (33). The clinical application of Treg cell therapy for autoimmune diseases such as type 1 diabetes, multiple sclerosis, and systemic lupus erythematosus (SLE) is promising, considering that ongoing clinical studies have shown some clinical benefit (34). However, additional research is required to determine whether the decreased number of Tregs in the peripheral blood contributes to the pathogenesis, as conflicting results were reported (7). These studies included a large proportion of AChR-positive MG patients, and the frequency of Tregs was negatively correlated with clinical severity (8, 9). In this regard, our study showed that Treg frequency was not significantly reduced in patients with AChR-positive MG than in HCs. In contrast, the refractory seronegative patients with MG in our study had a significantly decreased Treg frequency than HCs, and the decrease in MGC by IVIg was correlated with the frequency of Tregs.

The present study demonstrated that in a population of patients with refractory seronegative MG, Treg frequency decreases and correlates with disease severity. In addition, several studies have reported a decreased suppressive function of Tregs (35, 36). As a mechanism of action of IVIg treatment for MG, it is reported that IVIg treatment expanded CTLA4^+^ Tregs by modulating antigen presenting DC, since CTLA4^+^ Tregs were underrepresented and functionally defective in MG patients (37). However, in our study, neither AChR-positive patients nor patients with refractory seronegative MG had reduced Treg function compared to HCs. Notably, the other groups evaluated the function of Tregs immediately after isolation from donor blood, whereas we evaluated the function of Tregs expanded ex vivo for 13 days; this could explain the disparity in results. In addition to Tregs, T helper 17 cells (Th17) and follicular helper T cells (Tfh) are involved in the pathogenesis of MG (7). The number of Th17 cells and the level of IL-17A, which is mainly produced by the Th17 subset, in the peripheral blood of patients with MG correlate with the quantitative MG score (30, 38). Tfh are involved in the differentiation of B lymphocytes into plasma cells. Increased numbers of Tfh in the thymus and blood and increased levels of IL-21, a cytokine produced by Tfh, in the serum of patients with MG have been reported (39, 40). However, in our scRNA-seq analysis of PBMCs from patients with MG, it was not possible to distinguish between clusters of Th17 and Tfh based on their gene expression profiles. Furthermore, no significant changes in IL-17A or IL-21 levels were observed between patients with MG and HCs in the proteomic analysis of their plasma. Therefore, the involvement of Th17 or Tfh was not detected with the present scRNA-seq-based clustering method. Although Breg cell function is impaired in MG patients similar to Tregs, we did not identify a Breg cluster in our scRNA-seq data. This discrepancy arises because the previous publication utilized CD19^+^CD24^hi^CD38^hi^ as the Breg marker for FACS, whereas we classified immune cells based on gene expression profiles (41).

In this study, an increase in the frequency of memory B cells was observed in refractory seronegative MG compared with that in HC or AChR-positive MG groups, and the percentage of memory B cells was positively correlated with disease severity. Thus, memory B cells could contribute to the pathogenesis of refractory seronegative MG. Muto et al. (11) found that in two patients diagnosed with AChR-positive MG, there was an increase in the number of memory B cells in the blood several months before clinical relapse. In addition, Ruetsch-Chelli et al. (8) reported that in 34 patients with MG, including four considered seropositive for MG, the peripheral memory B-cell ratio was significantly higher in relapsed MG than that in MG that did not relapse. Similar to our study, Torres-Valles et al. also reported reduced B-cell counts in the patients diagnosed as Good’s syndrome (GS), a rare immunodeficiency by coexistence of a thymoma and hypogammaglobulinemia, and MG have also been frequently reported in GS patients (42). Rituximab treatment eliminates most CD20-positive B cells, including memory cells. However, a small number of CD20-positive B cells could survive and cause relapse by producing autoantibody-secreting B cells (19). In the previous studies performed by Jin et al. (10) and Jiang et al. (19), although the number of samples were relatively small compared to the present study, scRNA-seq was analyzed in n=2 and n=3 AChR-positive and Musk-positive MGs, respectively. In both analyses, the involvement of B cells in MG is strongly suggested. In the present study, AChR-positive MG (n=10) and seronegative MG (n=10 each before and after IVIg treatment) were analyzed, suggesting the involvement of B cells and Tregs. Taken together, seronegative MG could be a disease caused by autoantibodies similar to other types of MG, although the autoantigen responsible for the disease has not been identified. In terms of the involvement of other B-cell subtypes in seronegative MGs, previous studies have shown a reduction in plasmablast numbers in seronegative MG compared to those in HC and AChR-positive MG, whereas no difference was observed in the frequency of memory B cells (43). The discrepancies between our results and those in previous reports could be attributed to the differences in the disease severity of the patients analyzed. In this study, moderate or severe generalized MG was analyzed, whereas in previous studies, almost all the patients had mild generalized MG or milder symptoms.

Although the precise immunological mechanisms of various types of B cells have not been fully elucidated, some therapeutic strategies for the direct inhibition of B cells are administered for MG treatment (44). Among them, rituximab is recommended as an early therapeutic option for patients with MuSK-positive MG whose response to initial immunotherapy is inadequate (45). Rituximab is generally considered effective for refractory seronegative MG. Rituximab is not approved by the Japanese government for MG; therefore, we used IVIg therapy for seronegative MG. The mechanisms of action of IVIg in the treatment of MG remain poorly understood and are assumed to have no influence on the underlying immune cellular mechanisms. In fact, no changes in immunological, genetic, or proteomic parameters were detected before and after IVIg treatment. A comparison between before and after IVIg treatment was useful for establishing the association between these parameters and disease severity. Furthermore, we speculate that IVIg may shorten the half-life of pathogenic autoantibodies by saturating the anti-neonatal Fc receptor using excess normal IgGs.

The administration of immunosuppressants to AChR-positive MG patients decreased the naïve B-cell population while increasing the memory B-cell population (46). This study demonstrated changes in Treg and B-cell populations in refractory seronegative MG despite consistent administration of immunosuppressants before and after IVIg treatment. The correlation between improvement in disease severity and changes in B-cell and Treg frequency before and after IVIg treatment suggests that the results could be attributed to both immunosuppressants and the IVIg treatment. Thus, in refractory seronegative MG, where immune abnormalities cannot be detected by any examination, the evaluation of B-cell abnormalities should be emphasized for confirming immune abnormalities.

CD22 belongs to the sialic acid-binding immunoglobulin-like lectin (Siglec) family. It is present on the surface of mature B cells and, to a lesser extent, on some immature B cells (47). CD22 negatively regulates B-cell receptor signaling and plays a key part in establishing the B-cell activation threshold. Soluble CD22 is generated by the cleavage of the extracellular domain on the membrane surface and is a tumor marker for B-cell malignancies (48). In addition, CD22 is a therapeutic target in acute B-cell leukemia. The level of soluble CD22 is correlated with disease severity in patients with gram-negative bacterial sepsis (49). CD22 is a negative regulator of microglial phagocytosis in the brain. Inhibition of CD22 promotes microglial phagocytosis of Ab oligomers and lessens cognitive impairment in aged mice. The plasma levels of soluble CD22 are elevated in patients with Alzheimer’s disease, and its level was correlated with brain Ab burden, cerebrospinal fluid (CSF) p-tau levels, and baseline cognitive impairment (50). Therefore, increased levels of soluble CD22 are promising biomarkers of various disorders, including cancer, infections, and neurogenerative disorders.

In this study, plasma proteomic analysis revealed that the plasma levels of soluble CD22 decreased between refractory seronegative MG and HC or AChR-positive MG and correlated significantly with disease severity, immune cell frequency, and mRNA expression levels. We emphasize that the decreased levels of soluble CD22 were associated with the disease severity of refractory seronegative MG. We estimated that the consumption of CD22, a negative regulator of B-cell signal activation, especially in memory B cells, resulted in decreased levels of soluble CD22.

Quantitative deficiency of CD22 could be associated with the failure of B-cell activation, resulting in refractory courses in patients with seronegative MG. CD22 deficiency promotes the development of autoimmunity in autoimmune-susceptible mice and significantly increases autoantibody production, even in mice with partially downregulated CD22 expression (heterozygous CD22^+^/^−^ mice) (51). CD22 levels decrease in the B cells of patients with SLE who have active disease (52). In addition, there is a correlation between disease improvement following treatment and increased CD22 expression (53). Therefore, both increased and decreased levels of sCD22 in plasma are possible biomarkers for clinical practice.

This study has certain limitations. First, there were marked differences in disease severity and therapeutic profiles between patients with AChR-positive MG and those with refractory seronegative MG. The determination of refractory MG is based on therapeutic responses following the combination of several immunotherapies; therefore, it is difficult to obtain immunotherapy-free clinical samples. Second, although this study carried out one of the largest analyses of refractory seronegative MG and the preliminary results are of clinical significance, the study cohort, n = 10/group, is quite small. We have a clinical study in the pipeline to identify the possible biomarkers for soluble CD22 levels in more samples MG patients, using commercially available ELISA. We only evaluated early-onset female patients with refractory seronegative MG, not those with ocular MG or males with seronegative MG. Therefore, future studies should include a larger number of patients with various MG subsets. In addition, we should evaluate whether the age of patients influences the levels of soluble CD22. Third, we defined seronegative MG based solely on conventional radioimmunoprecipitation assays. Live cell-based assays can detect additional antibodies to clustered AChR, MuSK, and LRP4, resulting in higher sensitivity in patients with seronegative MG (54, 55). Autoantibodies were not evaluated using a live cell-based assay; therefore, seronegative MG patients may have very low levels of AChR, MuSK, and LRP4 autoantibodies.

In conclusion, the current findings contribute to a better understanding of the pathogenesis of MG and offer a future strategy for developing treatment options based on B-cell biology.

## Data availability statement

The scRNA-seq data that support the findings of this study have been deposited in GEO with the GSE227835 accession number (https://www.ncbi.nlm.nih.gov/geo/query/acc.cgi?acc=GSE227835). The plasma proteome data supporting the findings of this study are available in **Supplementary Table 2**.

## Ethics statement

The studies involving humans were approved by Institutional Review Board of Keio University #2020-0074. The studies were conducted in accordance with the local legislation and institutional requirements. The participants provided their written informed consent to participate in this study. Written informed consent was obtained from the individual(s) for the publication of any potentially identifiable images or data included in this article.

## Author contributions

YO: Writing – original draft. SM: Writing – review & editing. TI: Writing – review & editing. MS: Writing – review & editing. MI: Writing – review & editing. KI: Writing – review & editing. KS: Writing – review & editing. HM: Writing – review & editing. MO: Writing – review & editing. DW: Writing – review & editing. YK: Writing – review & editing. SS: Writing – review & editing, Writing – original draft, Visualization, Resources, Project administration, Methodology, Investigation, Funding acquisition, Formal analysis, Data curation, Conceptualization.
