# Supplementary material for: B-cell immune dysregulation with low soluble CD22 levels in refractory seronegative myasthenia gravis
Source: Front Immunol. 2024 Apr 22;15:1382320. doi: 10.3389/fimmu.2024.1382320 (PMC11071663; doi:10.3389/fimmu.2024.1382320)
Supplement: Supplementary file 1 [file DataSheet_1.docx]

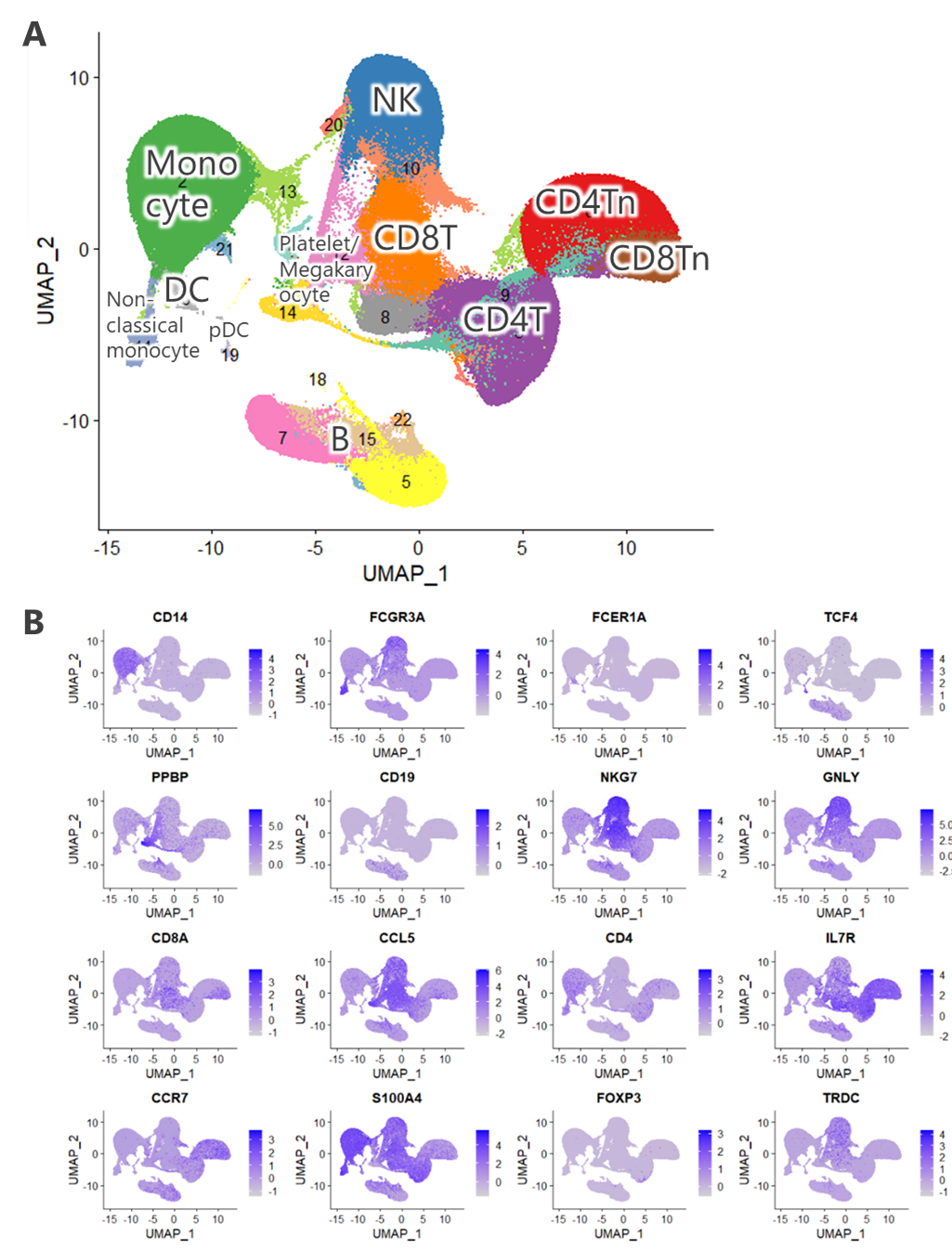


**Supplementary figure 1. Identification of major cell types**

**A**. uniform manifold approximation and projection (UMAP) showing identified 13 cell types - monocytes, non-classical monocytes, DCs, pDCs, B-cells, naïve CD4T-cells, CD4T-cells, Tregs, naïve CD8T-cells, CD8Ts, NK cells, platelets, and unclassified cells. B. Each UMAP showing gene expression levels of cell-type marker genes.


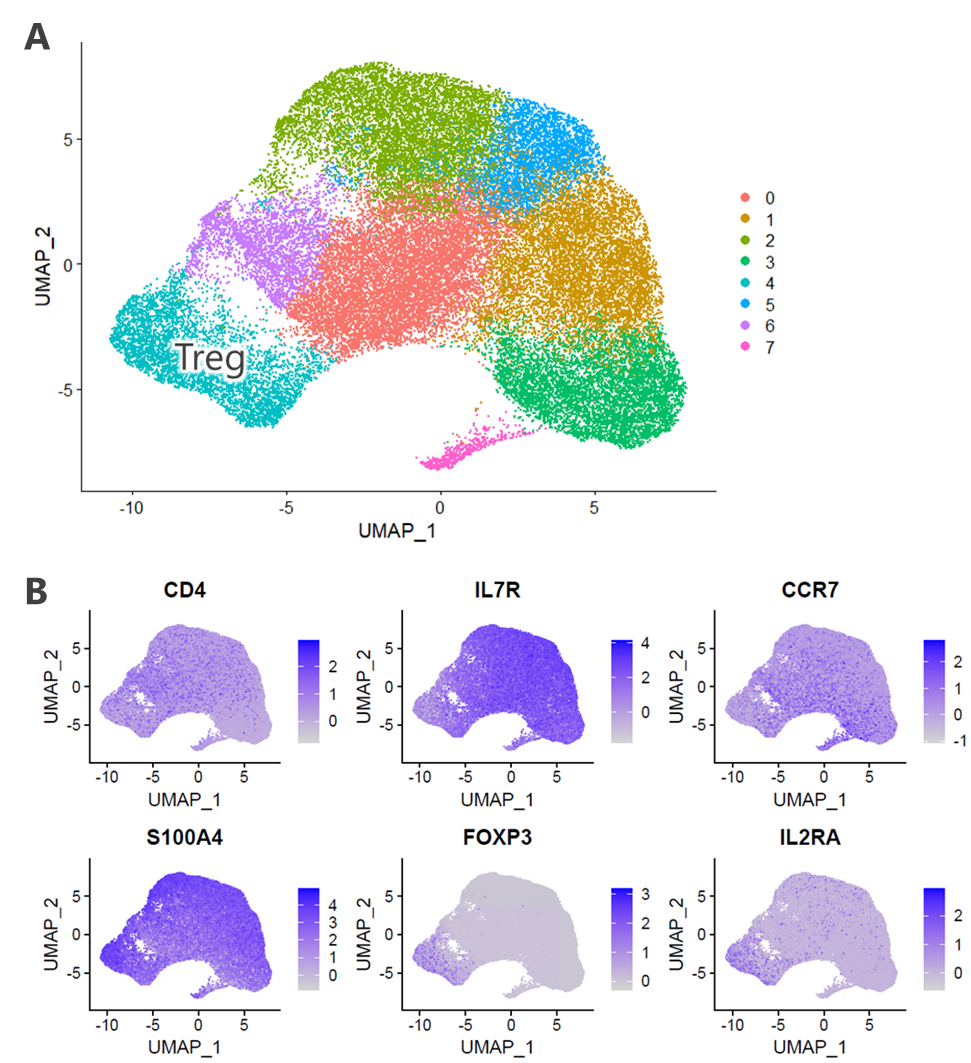


**Supplementary figure 2. Identification of Tregs in CD4T-cells**

**A**. the uniform manifold approximation and projection (UMAP) showing identified clusters by Louvain algorithm in CD4T-cells. B. Each UMAP showing gene expression levels of CD4T-cells and Treg related marker genes.


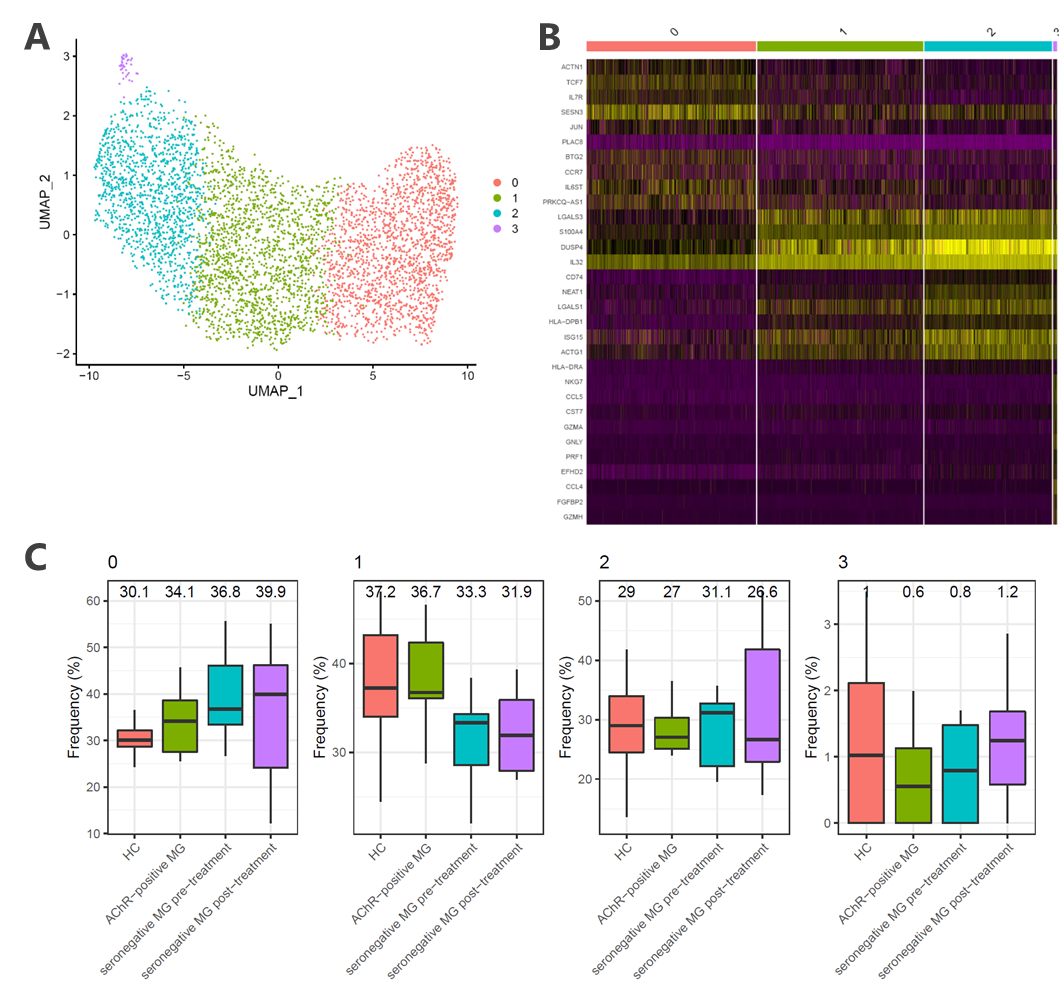

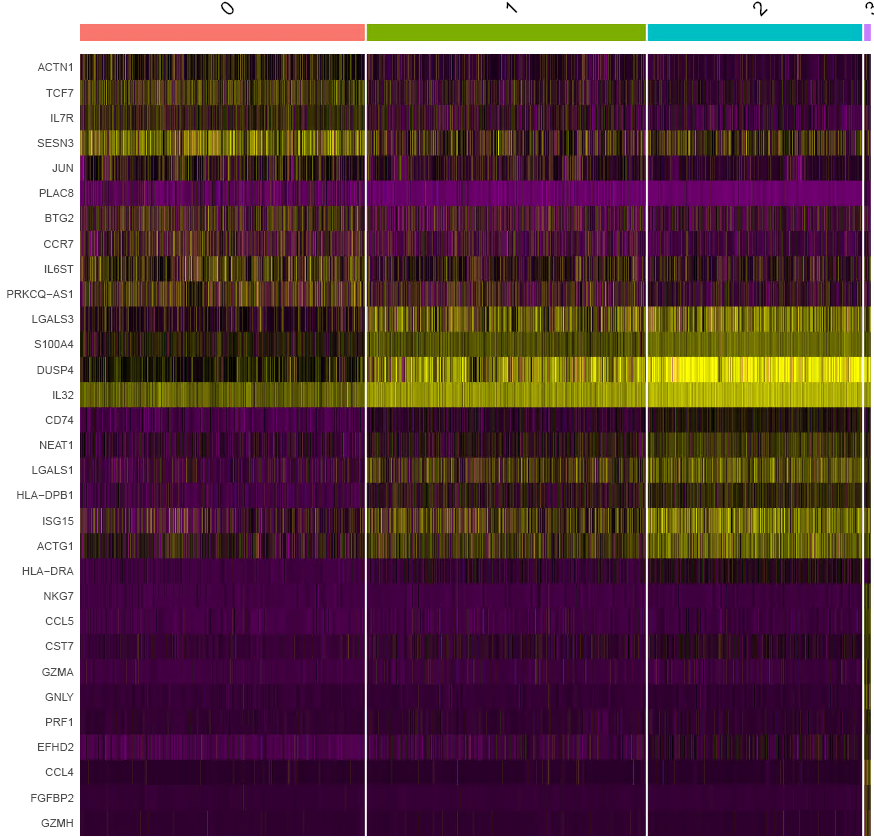


**Supplementary figure 3. Subpopulation analysis of Tregs**

**A**. the UMAP showing identified clusters by Louvain algorithm in Tregs. **B**. the heatmap showing diffrential expressed genes between each cluster of top 10 for each cluster. **C**. the frequencies of each cell type for each samples in Treg clusters. The frequency was displayed as the percentage of each cell types when the total number of cells in each sample was 100%.

**
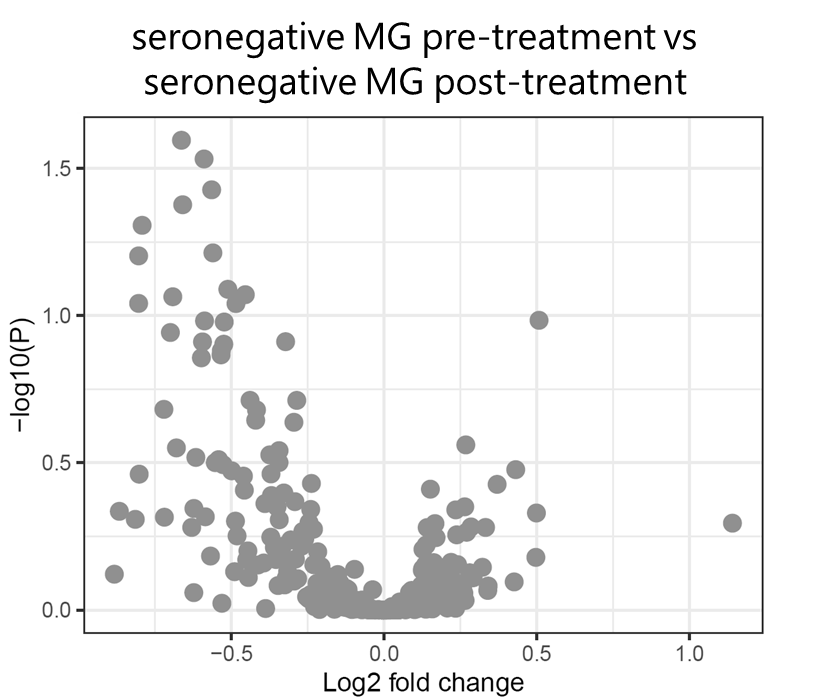
**

**Supplementary figure 4. Volcano plot of differentially expressed analysis between seronegative MG pre vs post-treatment**

The volcano plot showing -log10 P-values and log2 fold changes from differentially expressed analysis between each combination.
